# Molecular investigation of how drought stress affects chlorophyll metabolism and photosynthesis in leaves of C3 and C4 plant species: A transcriptome meta-analysis

**DOI:** 10.1016/j.heliyon.2025.e42368

**Published:** 2025-01-29

**Authors:** Shima Karami, Behrouz Shiran, Rudabeh Ravash

**Affiliations:** a Department of Plant Breeding and Biotechnology, Faculty of Agriculture, Shahrekord University, Shahrekord, Iran; b Institute of Biotechnology, Shahrekord University, P.O. Box 115, Shahrekord, Iran

**Keywords:** Photosynthesis, C3, C4, Drought, Meta-analysis

## Abstract

Drought stress has a significant impact on photosynthesis in plants, leading to reduced photosynthesis rates and affecting plant growth and yield. Understanding the effects of drought stress on photosynthetic pathways, particularly in C3 and C4 plants, is crucial for maximizing agricultural productivity and maintaining food security. In this study, we analyzed RNA-seq data from leaves of common wheat (*Triticum aestivum*) and sorghum (*Sorghum bicolor*), as representatives of C3 and C4, using a meta-analysis approach to investigate the photosynthesis-related genes involved in the response to drought stress. We identified specific genes and components of the photosynthesis pathway that are affected by drought stress. The findings suggest that wheat and sorghum respond differently to drought stress, with sorghum showing a more effective defense system against photoinhibition and damage to photosystems. On the other hand, it seems that in wheat, in order to deal with oxidative stress, the expression of homologous genes of C4 enzyme and genes involved in heme and siroheme synthesis pathway has increased under stress. This is probably due to the higher photoinhibition in C3 photosynthetic system compared to C4. Furthermore, drought stress affected chlorophyll biosynthesis and degradation pathways in both wheat and sorghum, but compared with sorghum, drought stress had a greater inhibitory effect on chlorophyll biosynthesis in wheat, which indicates the difference in their ability to cope with photoinhibition.

## 1 Introduction

Plants face numerous environmental stresses throughout their lifecycle, both in natural habitats and agricultural settings. Among these, drought is considered one of the most severe environmental factors that negatively impacts plant productivity, leading to a greater reduction in crop yield compared to other biological factors [1,2].

Photosynthesis is one of the main plant processes that is affected by drought stress. The susceptibility of photosynthesis-related processes to drought stress is primarily due to stomatal closure, resulting in reduced CO_2_ intake. This reduction directly affects the rate of photosynthesis, ultimately impacting plant growth and yield. Thus, the maintenance of photosynthetic rates during drought stress is crucial for plants to develop drought tolerance [3,4]. Plants have developed three different photosynthetic pathways, namely C3, C4, and crassulacean acid metabolism (CAM), to assimilate atmospheric CO_2_. In C3 plants, stomata are open during the day to absorb and fix CO_2_ and closed at night. The efficiency of this mechanism is low when C3 plants are subject to water limitation because they are unable to maintain their moisture under drought stress. C4 plants have developed a metabolic pump to concentrate CO_2_ in the bundle sheath cells, so CO_2_ fixation occurs in mesophyll cells and bundle sheath cells separately. This specific mechanism contributes to the higher water use efficiency of these plants compared to C3 plants and provides a better chance for C4 plants to survive in arid regions [5]. Differences in these pathways are expected to result in diverse reactions when plants are exposed to stress conditions. Some researchers have compared the responses of C3 and C4 plants to abiotic stresses such as drought and salinity [[6], [7], [8], [9]]. The role of genes related to photosynthesis as hub genes in the response to drought stress in both C3 and C4 plants has been highlighted [10]. In recent decades, the use of C4 photosynthesis-related key enzyme-encoding genes to increase the stress tolerance and productivity of C3 plants has become an important area of study in the genetic improvement of C3 plants [11]. However, previous attempts have been limited due to the complexity of C4 photosynthesis and the need for cell-type-specific expression and differential gene expression [12]. Considering the importance of in-depth investigation of photosynthetic metabolism under stress conditions and the lack of sufficient information on comparing of C3 and C4 plants response to drought stress, especially in terms of photosynthetic mechanisms, it highlights the necessity of studying the effects of drought on photosynthetic pathways and their molecular regulation networks. Therefore, the present study aimed to provide a better understanding of the impact of drought stress on photosynthetic metabolism in wheat and sorghum as representatives of C3 and C4 plants. This effort can be useful in developing strategies to improve photosynthesis as a tool for engineering drought tolerance and better managing drought stress in crop plants.

RNA-seq is a highly effective technique for measuring gene expression and transcription activation at the genome-wide level. Due to the high cost of sequencing, RNA-seq experiments are often conducted with a small number of biological replicates. The limited power of these studies, combined with technical and biological variability, can result in issues with reproducibility and cross-validation. To address these issues, meta-analysis can be employed to eliminate false-positive findings related to experimental and design conditions, thereby enhancing the reliability of research findings. Additionally, integrating data from multiple experiments can provide a more comprehensive understanding of biological processes compared to analyzing individual studies alone [13,14]. Due to the availability of microarray and RNA-seq data from various studies, meta-analysis methods are currently used in gene expression studies in various research fields, including studies on the response of plants to biotic and abiotic stresses. Bioinformatics (dry lab) analysis, often referred to as the in-silico portion of molecular research, plays a crucial role in ensuring the accuracy of results obtained from various molecular approaches [15,16]. Hence, in this study, we attempted to obtain more accurate results and comprehensive knowledge of the effects of drought stress on genes related to the C3 and C4 photosynthetic pathways by combining RNA-seq data using an in-silico transcriptome meta-analysis approach.

## 2 Materials and methods

### 2.1 RNA-seq dataset collection

To obtain expression data for in-silico transcriptome meta-analysis (in dry lab), we first explored the NCBI Sequence Read Archive (SRA) (https://www.ncbi.nlm.nih.gov/sra) and European Nucleotide Archive (ENA) database (https://www.ebi.ac.uk/ena/browser/home). To select studies focused on drought stress using leaf tissue samples from both wheat and sorghum plants, a search was conducted using keywords such as “water stress”, “drought stress”, “abiotic stress”, combined with the organism (common wheat and sorghum). Since wheat and sorghum are important cereals in the world and have different photosynthetic mechanisms (C3 and C4), they were considered for this study. Throughout the search process, several factors were considered, including the specific tissue type of interest (leaf), the inclusion of both control and drought treatment groups in the samples, the requirement for RNA-seq gene expression profile dataset, and the utilization of the Illumina HiSeq sequencing platform for RNA-seq. Then, four datasets (BioProject: PRJNA391522, PRJNA257938, PRJNA276167, and PRJNA809121) were selected and their RNA-seq raw reads were downloaded from the European Nucleotide Archive database (https://www.ebi.ac.uk/ena/browser/home).

### 2.2 Preprocessing and meta-analysis

Each dataset was processed with the following methods: First, the raw data quality was assessed using the FastQC tool (https://www.bioinformatics.babraham.ac.uk/projects/fastqc) [17]. Next, the clean data was obtained by removing low-quality and adapter sequences using the Trimmomatic tool [18]. To analyze the clean reads, they were mapped to the reference genome (http://ftp.ebi.ac.uk/ensemblgenomes/pub/release-49/plants/) of the corresponding plant using the STAR tool [19]. To normalize gene counts, the trimmed mean of M-values (TMM) method was employed. The EdgeR package [20] was utilized to determine differentially expressed genes (DEGs) in individual datasets. This analysis was conducted based on the Benjamini-Hochberg false discovery rate (FDR) with a significance threshold of 0.05. For the meta-analysis, the metaRNASeq R package was used [21]. This involved combining the p-values obtained from EdgeR using the Fisher method. Finally, only genes with a log2-fold change |log2FC|>1 and an adjusted p-value< 0.05, were selected as DEGs.

### 2.3 Functional enrichment analysis

Functional enrichment analysis was conducted to gain insights into the biological context of the meta-DEGs. The ShinyGO tool version 0.76 with default settings [22] was employed to perform gene ontology (GO) analysis, which encompassed molecular function (MF), biological process (BP), and cellular component (CC) terms. Additionally, pathway analysis was carried out using the Kyoto Encyclopedia of Genes and Genomes (KEGG) database. However, due to the unavailability of a wheat dataset, *Arabidopsis thaliana* was utilized instead to identify significantly enriched pathways.

### 2.4 Promoter motif analysis of photosynthesis related DEGs

To identify conserved motifs in photosynthesis-associated DEGs promoter sequences, we extracted the 1 kbp upstream regions of DEGs from Ensembl Plants (http://plants.ensembl.org) and examined them using MEME (https://meme-suite.org/meme/tools/meme) with default settings, although the maximum number of motifs was set to 10. To define the known motifs, the identified motifs were then compared to plant *cis*-acting regulatory elements in the PLACE database or *A. thaliana* DAP motif database using the Tomtom tool version 5.5.5 (http://meme-suite.org/tools/tomtom). The tool applied a threshold E-value<0.05 to determine the significance of motif matches. Additionally, we employed the GoMo tool Version 5.5.5 to uncover potential biological roles associated with these motifs. GoMo scans all promoters using nucleotide motifs provided by the user to determine if any motif is significantly associated with genes linked to one or more GO terms (http://meme-suite.org/tools/gomo).

### 2.5 Protein-protein interaction and identification of hub genes

Protein-protein interaction (PPI) network of DEGs related to photosynthesis was performed using STRING database (version 12) (https://string-db.org/). The protein sequence of DEGs obtained from Ensembl Plants was used as input data to construct PPI string networks. In addition, by Cyto-Hubba plugin of Cytoscape software [23], hub genes were identified in each network.

**Fig. 1:**
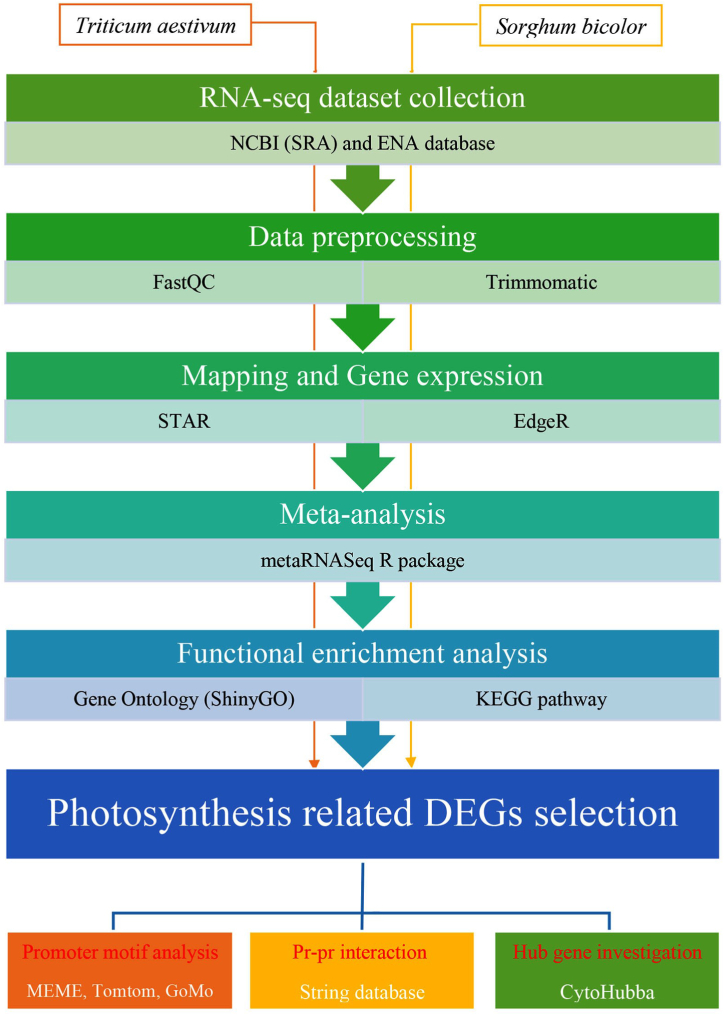
The step-by-step schematic workflow of meta-analysis and bioinformatic analysis.

The step-by-step schematic workflow of meta-analysis and bioinformatic analysis used in this study to investigate the effect of drought stress on the expression of genes involved in chlorophyll metabolism and photosynthesis is presented in Fig. 1.

## 3 Results

### 3.1 Meta-analysis and functional enrichment analysis

To investigate the impact of drought stress on the expression profiles of genes involved in the photosynthesis pathway of C3 and C4 representatives (wheat and sorghum) and compare their responses, we conducted a meta-analysis using RNA-seq studies. The output of a meta-analysis study is a list of genes that are expressed across different studies. Our findings revealed that a significant number of genes were differentially expressed in both sorghum (C4) and wheat (C3). Specifically, 2524 genes in sorghum and 14656 genes in wheat exhibited differential expression (adj p-value ≤0.05). Obtaining a list of DEGs is only the starting point for gaining biological insight into the subject of study. To understand the biological basis of DEGs, functional enrichment analysis is performed that relies on annotation databases such as GO and KEGG [24].

**Fig. 2:**
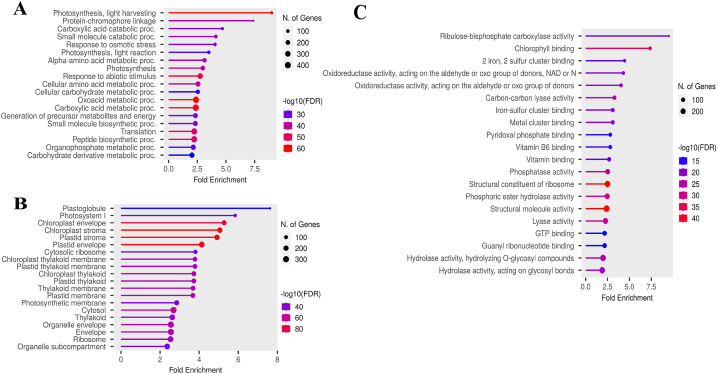
**Enriched GO terms in wheat under drought stress**. Biological processes (A), cellular components (B), and molecular functions (C).

**Fig. 3:**
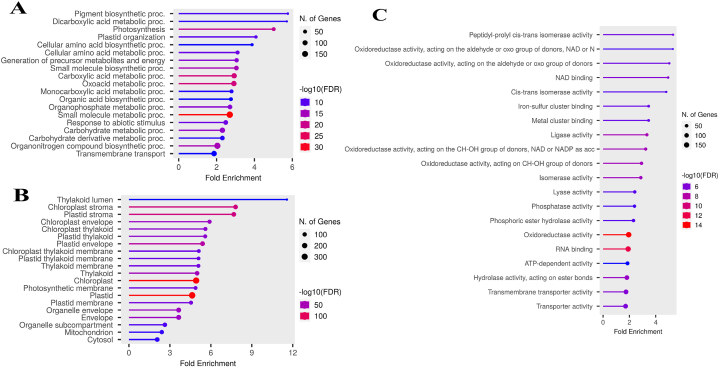
**Enriched GO terms in sorghum under drought stress**. Biological processes (A), cellular components (B), and molecular functions (C).

GO enrichment analysis is one of the most widely used methods to determine the molecular function, cellular components, and biological process of a gene [25]. Our GO results in wheat and sorghum were remarkable, as GO terms associated with photosynthesis ranked among the top 20 terms in each biological process, cellular components, and molecular function categories (Fig. 2, Fig. 3A–C). Some of the key terms related to wheat included photosynthesis, light harvesting, light reaction, protein-chromophore linkage, generation of precursor metabolites and energy in the category of biological processes, Ribulose-bisphosphate carboxylase activity, chlorophyll (Chl) binding, metal cluster binding, 2-iron 2-sulfur cluster binding in the molecular function category and photosystem I, chloroplast envelope, chloroplast stroma, chloroplast thylakoid membrane, photosynthetic membrane in the cellular component category (Fig. 2A–C). The GO analysis results for sorghum revealed that the majority of the top 20 GO terms in the biological processes category were directly associated with photosynthesis. These terms included photosynthesis, pigment biosynthetic process, plastid organization, and generation of precursor metabolites and energy (Fig. 3A). Additionally, in the cellular component category, terms such as thylakoid lumen, chloroplast stroma, chloroplast thylakoid membrane, chloroplast envelope, plastid thylakoid membrane, and photosynthetic membrane ranked among the top 20 terms (Fig. 3B). This highlights the crucial role of photosynthesis in response to drought stress in both C3 and C4 plants.

KEGG pathway enrichment analysis has paved the way for identifying and screening active biological metabolic pathways in plants and clarifying metabolic mechanisms activated in response to drought [26]. KEGG pathway analysis of wheat and sorghum plants revealed a significant enrichment of DEGs in metabolic pathways associated with photosynthetic processes. These pathways, included photosynthesis, photosynthesis-antenna proteins, carbon fixation in photosynthetic organisms, and porphyrin and Chl metabolism. In wheat, 283 DEGs were related to these pathways, with 38 related to photosynthesis, 78 related to the light-harvesting Chl protein complex, 98 to carbon fixation, and 69 to porphyrin and Chl metabolism (Table S1). In sorghum, 51 DEGs were associated with these pathways, with 9 related to photosynthesis, 2 to the light-harvesting Chl protein complex, 28 to carbon fixation, and 12 to porphyrin and Chl metabolism (Table S2).

### 3.2 DEGs related to photosynthesis and antenna proteins

**Fig. 4:**
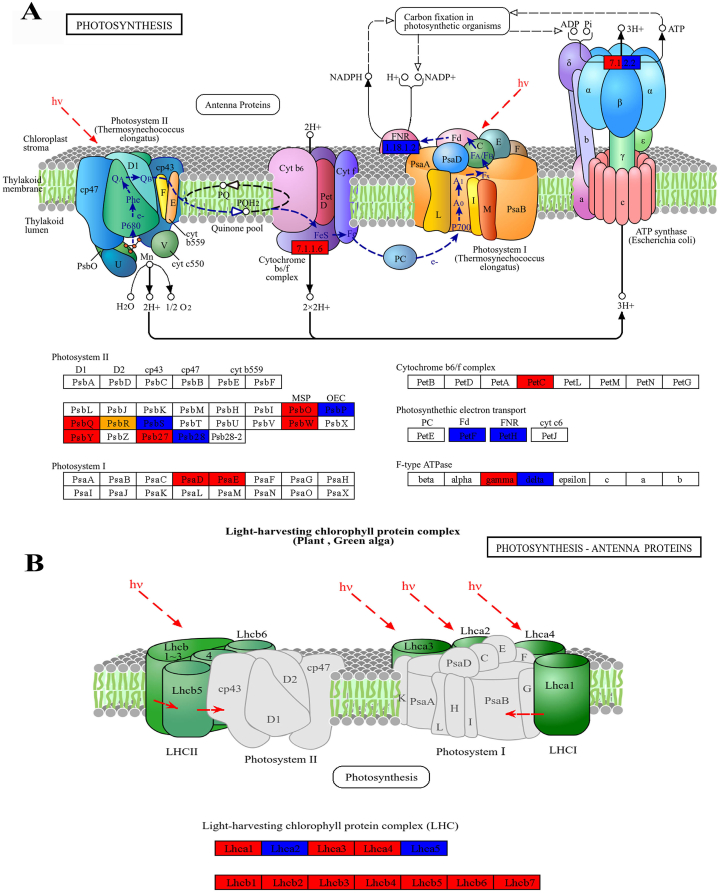
**Differentially expressed genes involved in the photosynthesis pathway (A) and photosynthesis-antenna proteins (B) in wheat and sorghum under drought stress**. The blue, red, and orange boxes indicate the genes that are commonly expressed in both wheat and sorghum, exclusively in wheat, and exclusively in sorghum, respectively.

The findings revealed that genes associated with different parts of the photosynthesis pathway, including photosystem I (PSI), photosystem II (PSII), light-harvesting complex (LHC) protein, cytochrome *b*6f complex (Cyt *b*_*6*_*f*), and ATP synthase, were affected by drought stress and their expression was significantly decreased (Table S1 and Table S2). Interestingly, genes related to the PSI reaction center subunit showed significant expression changes only in wheat, such as *PSI reaction center subunit II* (*PsaD*) and *PSI reaction center subunit IV* (*PsaE*). In contrast to PSI, genes responsible for PSII reaction center subunit were affected by drought in both wheat and sorghum. PSII-related DEGs that were common in both plant groups included genes encoding oxygen-enhancing protein 2 (*PsbP*), PSII protein 22 kDa (*PsbS*) and PSII reaction center protein Psb28 (*Psb28*). In addition, genes encoding various subunits of the PSII protein supercomplex, namely, oxygen-evolving enhancer protein (PsbO), PSII repair protein PSB27 (Psb27), Oxygen-evolving enhancer protein (PsbQ), PSII reaction center W (PsbW), and PSII reaction center protein Y (PsbY), which play crucial roles in the light reaction, were down-regulated only in wheat and remained unchanged in sorghum (Fig. 4A). Furthermore, the findings indicated that drought stress led to the down-regulation of LHC proteins in both wheat and sorghum. However, the expression of genes related to LHC was more affected in wheat than in sorghum. In sorghum, only the *Lhca5* and *Lhca6* were differentially expressed, whereas in wheat, all LHC-related genes including *Lhca1-Lhca6* and *Lhcb1*-*Lhcb7* were differentially expressed (Fig. 4B). Apart from the light-harvesting antenna proteins, the expression of genes related to the Cyt *b*_*6*_*f* protein complex and f-type ATPase subunits, as well as the *ferredoxin* (*Fd*) and *ferredoxin NADP* + *reductase* (*FNR*) involved in electron transport, significantly decreased under stress in wheat. Conversely, in sorghum, the expression of the gene encoding the subunit of the Cyt *b*_*6*_*f* complex did not significantly change, and the expression of the *Fd* showed the opposite pattern compared to that in wheat.

In plants, there are two important pathways for cyclic electron flow (CEF). The first pathway involves the proton gradient regulation 5 (PGR5)/PGR5-like 1 (PGRL1) protein and the second pathway involves the chloroplast NADH dehydrogenase-like complex (NDH) components [27]. Results in wheat showed that drought stress significantly reduced the expression of genes involved in the two CEF pathways. So that the expression of four genes related to the PGR5/PGRL1-dependent CEF pathway (*PGRL1*) and 16 genes related to the NDH complex, including genes encoding NDH subunits (*ndhN*, *ndhO*, and *ndhT*), *PHOTOSYNTHETIC NDH SUBUNIT OF SUBCOMPLEX B 3* (*PnsB3*) and *PnsL*, was down-regulated. While in sorghum, the expression of CEF-related genes remained unchanged except for *PGRL1* which was upregulated (Table 1). These findings indicate that the response to drought stress differs between wheat and sorghum, particularly in terms of CEF-related gene expression.

**Table 1:** Differentially expressed genes associated with in cyclic electron flow in wheat and sorghum under drought stress.

plant	Gene ID	FDR-pValue	logFC	Arabidopsis gene ID	Gene name
**Wheat**	TraesCS3A02G407200	3.03E-05	−1.19559	AT5G58260	*ndhN*
	TraesCS3B02G441000	1.69E-05	−1.16352	AT5G58260	*ndhN*
	TraesCS3D02G402500	0.002379	−0.84676	AT5G58260	*ndhN*
	TraesCS3A02G502300	6.55E-06	−1.05119	AT1G74880	*ndhO*
	TraesCS3D02G509100	0.001451	−1.3458	AT1G74880	*ndhO*
	TraesCS4A02G203400	4.65E-27	−2.81683	AT4G09350	*ndhT*
	TraesCS4B02G107300	4.29E-15	−2.33956	AT4G09350	*ndhT*
	TraesCS4D02G104200	2.65E-23	−3.22937	AT4G09350	*ndhT*
	TraesCS1D02G031700	8.15E-07	−0.85518	AT5G59400	*PGRL1*
	TraesCS4A02G319000	2.31E-12	−1.23172	AT4G22890	*PGRL1*
	TraesCS5B02G560400	7.9E-08	−0.73355	AT4G22890	*PGRL1*
	TraesCS5D02G569800	6.55E-15	−1.05117	AT4G11960	*PGRL1*
	TraesCS4A02G232300	4.81E-05	−1.1132	AT1G15980	*PNSB1*
	TraesCS4B02G083600	0.006634	−0.87734	AT1G15980	*PNSB1*
	TraesCS4D02G081600	0.007426	−1.07037	AT1G15980	*PNSB1*
	TraesCS7A02G108600	0.004438	−1.09253	AT3G16250	*PNSB3*
	TraesCS7B02G006600	5.12E-05	−1.00659	AT3G16250	*PNSB3*
	TraesCS7D02G103500	8E-18	−1.18614	AT3G16250	*PNSB3*
	TraesCS6B02G331700	1.54E-05	−1.25988	AT4G39710	*PNSL4*
	TraesCS1B02G011100	0.000941	−0.96152	AT5G13120	*PNSL5*
**Sorghum**	SORBI_3009G011600	1.04E-10	1.095123472	AT5G59400	*PGRL1*

### 3.3 DEGs related to carbon fixation

The results showed the impact of drought stress on the expression patterns of genes involved in carbon fixation in both wheat and sorghum plants (Table S1 and Table S2). The majority of genes encoding crucial enzymes in the Calvin cycle, such as ribulose bisphosphate carboxylase small subunit (RBCS), triosephosphate isomerase (TIM), phosphoribulokinase (PRK), fructose-bisphosphate aldolase (FBA), fructose-1,6-bisphosphatase 1 (FBP), d-ribulose-5 phosphate 3-epimerase (RPE), and chloroplastic photosynthetic glyceraldehyde-3-phosphate dehydrogenase (GAPDH), were downregulated. Furthermore, alterations in the expression of key enzymes in the C4 photosynthesis pathway, such as NADP-malic enzyme (NADP-ME), and phosphate dikinase (PPDK), were observed in both wheat and sorghum. Interestingly, the *malate dehydrogenase* (*MDH*) gene, another important enzyme in the C4 photosynthetic pathway, was upregulated in wheat, while its expression remained unchanged in sorghum.

### 3.4 DEGs related to porphyrin and chlorophyll metabolism

**Fig. 5:**
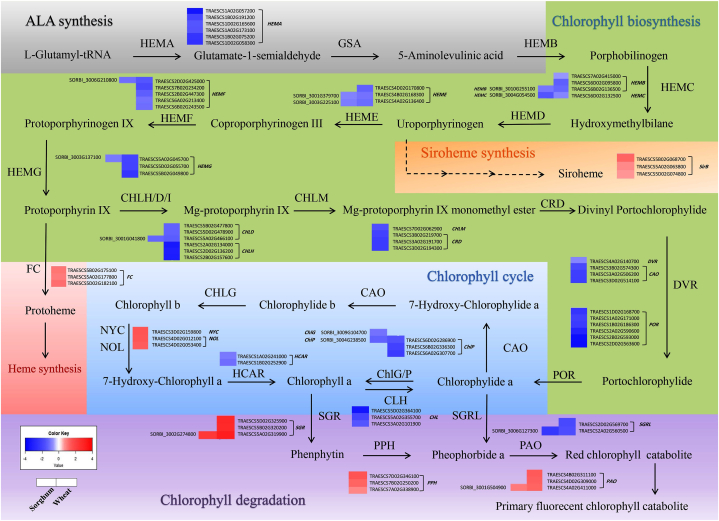
**Heatmap of differentially expressed genes involved in porphyrin and chlorophyll metabolism**. *HEMA*, Glutamyl-tRNA reductase; *GSA*, glutamate-1-semialdehyde aminotransferase; *HEMB*, 5-aminolevulinate dehydrogenase; *HEMC*, Porphobilinogen deaminase; *HEMD*, Uroporphyrinogen-III synthase; *HEME*, uroporphyrinogen III decarboxylase; *HEMF*, coproporphyrinogen III oxidase; *HEMG*, protoporphyrinogen oxidase; *CHLD*, magnesium chelatase subunit D; *CHLH*, magnesium chelatase subunit H; *CHLM*, Mg-protoporphyrin IX methyltransferase; *CRD*, Mg-protoporphyrin IX monomethyl ester oxidative cyclase; *DVR*, Divinyl chlorophyllide *a* 8-vinyl-reductase; *CAO*, Chlide *a* oxygenase; *ChlP*, geranylgeranyl diphosphate reductase; *ChlG*, Chl synthase; *NYC1*, non-yellow coloring1; *NOL*, NYC1-like; *HCAR*, hydroxymethyl chlorophyll *a* reductase; *CLH*, Chlorophyllase; *SGR*, stay-green; SGRL, SGR-like; *PPH*, pheophytinase; *PAO*, pheophorbide *a* oxygenase; *SirB*, sirohydrochlorin ferrochelatase; *FC*, ferrochelatase.

Tetrapyrrole biosynthesis is a crucial process in photosynthetic organisms because it produces essential molecules like Chl, heme, siroheme, and phytochromobilin. These tetrapyrroles are all derived from a shared biosynthetic pathway [28]. Analysis of the expression of genes related to chlorophyll metabolism in wheat and sorghum plants under drought stress revealed that, the expression levels of genes encoding key enzymes involved in Chl biosynthesis were significantly decreased (Fig. 5). These genes included *HEMB* (*5-aminolevulinate dehydrogenase*), *HEMC* (*porphobilinogen deaminase*), *HEME* (*uroporphyrinogen III decarboxylase*), and *HEMF* (*coproporphyrinogen III oxidase*), *HEMG* (*protoporphyrinogen oxidase*; PPO) and *CHLD* (*magnesium (Mg) chelatase subunit D*). Additionally, some other genes, including *glutamyl-tRNA reductase* (GluTR; *HEMA*), *Mg-protoporphyrin IX methyltransferase* (*CHLM*), *divinyl chlorophyllide a 8-vinyl-reductase* (*DVR*), and *Mg-protoporphyrin IX monomethyl ester (oxidative) cyclase* (*CRD*), were down-regulated in wheat and remained unchanged in sorghum. The *Uroporphyrinogen-III synthase* (*HEMD*) was not differentially expressed in both wheat and sorghum.

Chl metabolism is an intricate process that involves both the biosynthesis and degradation of Chl. Under normal conditions, the balance between these processes maintains a steady level of Chl. However, in response to environmental stresses that lead to the partial or complete dismantling of the photosynthetic machinery, Chls undergo turnover or breakdown [29,30]. In our research, we found that both wheat and sorghum plants exhibited differential expression of genes related to the Chl cycle and degradation when subjected to drought stress. For example, the *geranylgeranyl diphosphate reductase* (*ChlP*) was down-regulated in both plants. However, the genes encoding the chlide *a* oxygenase (*CAO*) enzyme and the genes encoding two isoforms of Chl *b* reductase, *non-yellow coloring1* (*NYC1*) and *NYC1-like* (*NOL*), which are involved in Chl cycling, were differentially expressed under stress in wheat but not in sorghum. On the other hand, genes encoding Chl synthase (*ChlG*) were differentially expressed only in sorghum. Among the genes related to the Chl degradation pathway, the up-regulated *pheophorbide a oxygenase* (*PAO*) and *STAY-GREEN* (*SGR*) genes and the down-regulated *SGR-like* (*SGRL*) gene were common in both plants. The expression of the gene encoding pheophytinase (*PPH*), another enzyme involved in chlorophyll degradation, changed only in wheat during drought stress. In general, similar patterns were observed in the expression of porphyrin and Chl metabolism pathway genes in wheat and sorghum, although more genes were differentially expressed in wheat than in sorghum.

### 3.5 Promoter motif analysis

To investigate the regulatory mechanisms affecting the expression of genes related to photosynthesis in wheat and sorghum under drought stress conditions, a promoter motif analysis was performed on 1 kbp upstream flanking regions of DEGs related to photosynthesis. In total, 10 conserved motifs with E-value <1e^−4^ and width from the range 10–50 bp were identified, which could play a role in the regulation of these genes. To search for known motifs, the 10 motifs identified in each plant were compared to the *A. thaliana* DAP motif database. Any motifs that did not have a significant match in the *A. thaliana* DAP motif database were compared to the PLACE database.

#### 3.5.1 Motif analysis in wheat

The results in wheat showed that the highly frequently occurring motifs 2, 3, 10, and 8 were found across 278, 190, 129, and 109 DEGs. These 4 motifs were matched to MYB63, AP2/B3-like transcription factor family protein, C2C2dof, and AP2EREBP, respectively (Table 2). The GoMo analysis indicated that the identified motifs were associated with significant GO-terms under BP, MF, and CC categories such as regulation of transcription: DNA-dependent under the BP category, and transcription factor activity and ATP binding under the MF category (Table 2).

**Table 2:** Motif analysis of differentially expressed genes in wheat.

motif	Motif Logo	E-value	Width	known motif	Significant GO-toms
1		6.80E-100	50	CIACADIANLELHC	CC chloroplast
2		1.90E-86	29	MYB63	CC nucleus CC mitochondrion CC chloroplast stroma MF transcription factor activity MF ATP binding
3		4.20E-64	29	ABI3VP1	MF transcription factor activity CC plasma membrane CC nucleus BP regulation of transcription, DNA-dependent
4		1.90E-55	41	ARR1AT	
5		4.00E-57	50	SORLIP1AT	CC chloroplast
6		5.60E-29	50	CGCGBOXAT	CC mitochondrion CC chloroplast stroma CC chloroplast envelope MF structural constituent of ribosome MF DNA-directed RNA polymerase activity
7		4.20E-21	50	10PEHVPSBD	
8		1.20E-18	29	AP2EREBP (ERF105)	
9		1.30E-16	21	FRS9	
10		6.40E-14	15	C2C2dof ABI3VP1	

#### 3.5.2 Motif analysis in sorghum

The result of promoter motif analysis in sorghum revealed that motifs 3 and 8 were the predominant motifs. These motifs were present in all the photosynthesis-related DEGs and were matched to AP2EREBP and C2C2dof (Table 3). The GoMo analysis showed that identified motifs were involved in the regulation of transcription, and translation, under the BP category, and transcription factor activity, RNA binding, nucleotide binding, and structural constituent of ribosome under the MF category (Table 3).

**Table 3:** Motif analysis of differentially expressed genes in sorghum.

motif	Motif Logo	E-value	Width	known motif	Significant GO-toms
1		1.50E-108	50	C2H2 (AtIDD11)	MF transcription factor activity CC endomembrane system BP regulation of transcription CC plasma membrane
2		5.50E-109	50	DOFCOREZM	CC endomembrane system MF transcription factor activity CC plasma membrane
3		1.50E-83	41	AP2EREBP (ESE1)	CC mitochondrion CC nucleus CC chloroplast envelope CC chloroplast stroma MF nucleotide binding
4		8.30E-51	21	ABI3VP1 (VRN1)	MF transcription factor activity CC plasma membrane CC nucleus BP regulation of transcription, DNA-dependent
5		1.20E-55	42		MF transcription factor activity CC endomembrane system
6		6.00E-31	41	AP2EREBP (ERF73)	CC mitochondrion CC chloroplast stroma CC chloroplast envelope MF RNA binding MF transcription factor activity
7		6.10E-18	27	SEF4MOTIFGM7S	CC endomembrane system
8		4.40E-10	11	C2C2dof	MF transcription factor activity BP regulation of transcription CC plasma membrane
9		1.40E-08	21	HMGBOX1	CC chloroplast thylakoid membrane CC mitochondrion
10		3.20E-05	21	AP2EREBP (SHN3)	CC chloroplast CC mitochondrion MF structural constituent of ribosome BP translation CC ribosome

This analysis provided insights into potential regulatory elements involved in the differential expression of photosynthesis-related genes in response to drought stress in wheat and sorghum.

### 3.6 Protein-protein interaction and identification of hub genes

**Fig. 6:**
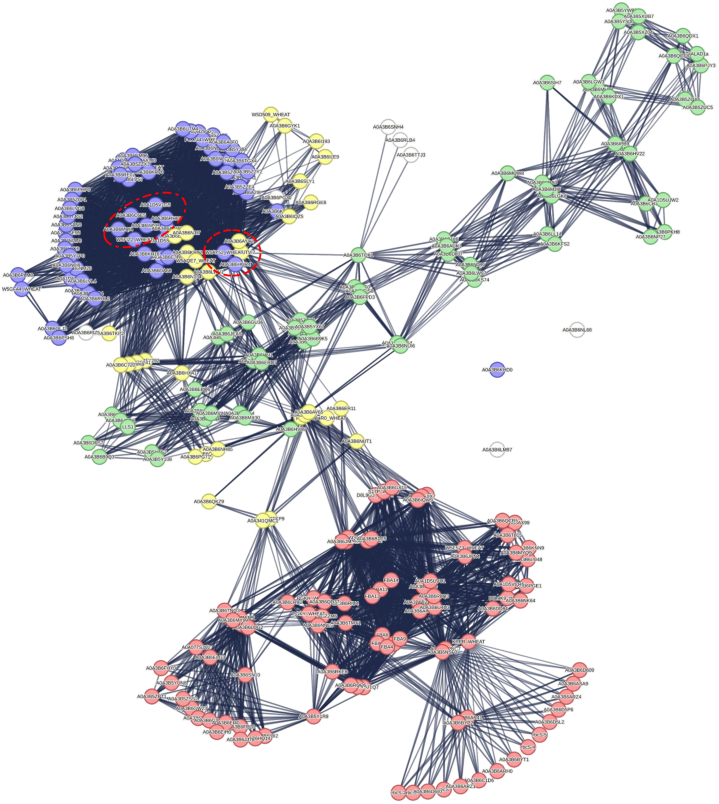
**PPI network analysis of photosynthesis-related DEGs in response to drought stress in wheat.** The dashed line indicates hub nodes and the color of the nodes indicates genes related to photosynthesis (yellow nodes), antenna proteins (blue nodes), carbon fixation in photosynthetic organisms (red nodes), and porphyrin and chlorophyll metabolism (green nodes).

**Fig. 7:**
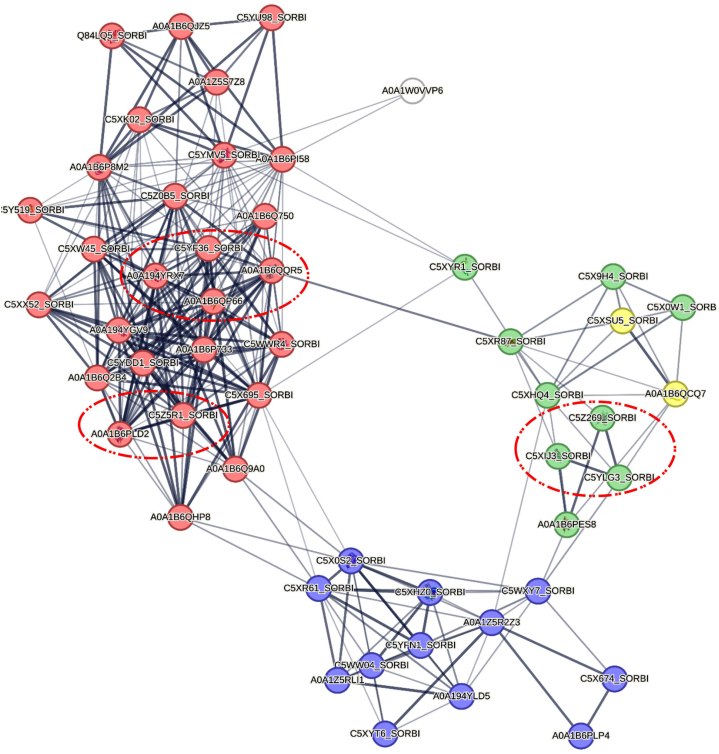
**PPI network analysis of photosynthesis-related DEGs in response to drought stress in sorghum.** The dashed line indicates hub nodes and the color of the nodes indicates genes related to Photosynthesis (green nodes), antenna proteins (yellow nodes), carbon fixation in photosynthetic organisms (red nodes), and porphyrin and chlorophyll metabolism (blue nodes).

**Fig. 8:**
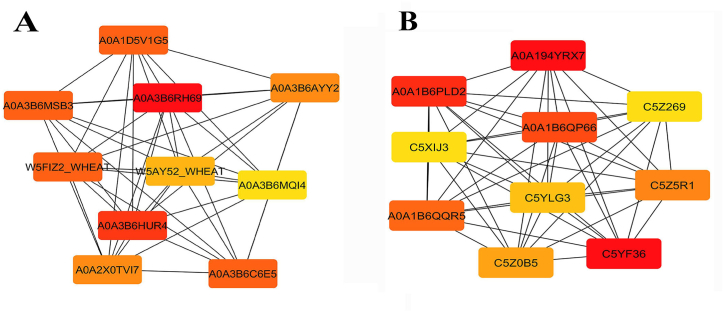
**subnetwork of top 10 nodes ranked by MCC scoring method using CytoHubba: Wheat (A) and Sorghum (B).** The color of the nodes indicates the degree of connectivity; red color indicates a higher degree.

Proteins and their functional interactions form the backbone of cellular processes. Protein-protein interaction networks can be effective in the complete understanding of biological phenomena. The STRING database is one of the best resources for examining protein-protein interaction networks [31]. In order to investigate the interaction network of photosynthetic proteins in response to drought stress, we analyzed 283 DEGs related to photosynthesis in wheat and 51 DEGs in sorghum using the STRING network database (Fig. 6, Fig. 7). To identify essential proteins in each PPI network, Cytohuba plugin was used. Top 10 essential nodes ranked by MCC scoring method were selected as hub genes in each network (Fig. 6, Fig. 7). The results showed that genes such as *LHCA* (A0A3B6RH69) and *LHCB* (A0A3B6HUR4) had the highest connectivity in wheat PPI network (Fig. 8A) and genes such as *PRK* (C5YF36_SORBI), *PRK* (A0A194YRX7) and *GAPDH* (A0A1B6PLD2) had the highest connectivity in sorghum PPI network (Fig. 8B).

## 4 Discussion

Photosynthesis is a vital process in plants and plays a key role in carbon fixation and the accumulation of biomass. It is crucial for the growth and development of plants. The process of photosynthesis can be divided into two main parts: light reactions and light-independent carbon reactions. Light reactions take place in the thylakoid membrane system and are responsible for producing ATP and NADPH. These energy-rich molecules are then utilized in light-independent carbon reactions (dark reactions), where atmospheric CO_2_ is converted into organic compounds [32].

### 4.1 Light reaction

The primary reactions of photosynthesis are initiated by the concentration of light energy into PSII and PSI. Photosystems are accompanied by LHCs, which play a crucial role in capturing sunlight in plants and green algae [33,34]. *Lhca1–Lhca6* are the six genes responsible for encoding the antennae of PSI, with Lhca1–4 being the primary components of the antenna system in higher plant photosystems and tightly integrated within the core complex of PSI. On the other hand, the antenna of PSII are encoded by *Lhcb1–Lhcb7* [35]. The study revealed that drought stress caused a decrease in the expression of light-harvesting Chl *a/b*-binding proteins in wheat and sorghum, with wheat being more significantly impacted compared to sorghum. Previous research has also shown that LHC-related genes are down-regulated under abiotic stress conditions such as drought [35,36].

PSI and PSII function consecutively in the photosynthetic energy transport chain and play a role in light-dependent carbon fixation reactions [37]. Different subsets of Chl molecules in these photosystems are excited by light energy, resulting in the transfer of electrons across a sequence of redox carriers known as the electron transfer chain (ETC). This process starts with the oxygen evolving complex (OEC) of PSII, which oxidizes water and releases oxygen and protons. The electrons then pass through the plastoquinone (PQ) pool, the cyt *b*_*6*_*f* complex, and plastocyanin (PC) before ultimately reaching PSI. In chloroplasts, PSI facilitates light-driven electron transport from PC to Fd, subsequently leading to the reduction of NADP^+^ to NADPH by FNR:NADP^+^ reductase. This process ultimately provides NADPH as a reducing power for the Calvin-Benson cycle and recycles NADP^+^ as an electron acceptor in PSI [38,39]. We found that the genes encoding these complexes were regulated under drought stress. The genes related to the OEC were down-regulated. PsbO is an essential component for the binding of PSII, and it assumes a significant role in maintaining the optimal activity of the OEC. Mutation in PsbO results in incomplete assembly of the PSII complex [40]. Defective functioning of the OEC and a lack of electron supply can be consequences of the down-regulation of *PsbQ* and *PsbP*, which disrupts the PSII reaction center. Additionally, an unstable OEC can lead to the generation of reactive oxygen species (ROS) [41]. The 10 kDa protein PsbR has been suggested to function as a docking protein for OECs in plants. Interestingly, the expression of the gene encoding PsbR increased in sorghum under stress, while no significant alterations were observed in wheat. PsbR, together with PsbQ, is involved in the organization of PSII components. In Arabidopsis, the absence of PsbR and PsbQ has been found to impair short-term adaptive mechanisms [42]. These findings emphasize the importance of PsbR and PsbQ in the organization and functioning of PSII in sorghum, particularly under stress conditions. The down-regulation of genes associated with PSI, such as *PsaD* which was observed in wheat in our study, can cause a decline in PSI activity. This decline disrupts the transfer of electrons from PSII to PSI, resulting in an increase in excess excitation energy, an elevation of ROS levels, and an exacerbation of damage to PSII [40].

Linear electron flow (LEF) in photosynthesis leads to the accumulation of protons in the thylakoid lumen, generating a proton motive force (pmf) across the thylakoid membrane [30]. Pmf consists of a proton (H^+^) gradient (ΔpH) and a membrane potential (Δψ), both of which are essential for driving ATP synthesis via chloroplast ATP synthase [43]. Along with NADPH, ATP plays a vital role in providing assimilation power and energy for CO_2_ fixation, as well as assisting plants in coping with abiotic stress [32,44]. In this study, the transcript levels of ATP synthase subunits, including delta (in both wheat and sorghum) and gamma (only in wheat), were decreased significantly under drought conditions. This suggests that drought stress may inhibit ATP synthesis, particularly in wheat. Stress-induced inhibition of ATP synthesis not only influences the supply of assimilation power but can also negatively affect Rubisco activity, ultimately impacting CO_2_ fixation in plants [44]. We also observed a decrease in the expression of genes related to Rubisco subunits in both groups of plants.

Under stress conditions, decreased Calvin cycle enzymatic activity leads to impaired NADPH usage and NADP recycling. This can overload LEF and cause ROS production, leading to photoinhibition of PSI and PSII [39]. Protecting against photoinhibition may aid plant adaptation [45]. Various photoprotective strategies have been identified to avoid photoinhibition, including the ability to cope with excessive light absorbed by photosynthetic pigments [46]. The ΔpH component of pmf plays a crucial role in regulating photosynthesis by reducing the efficiency of light capture through the energy-dependent quenching (qE) of nonphotochemical quenching (NPQ) mechanism and slowing electron transfer to prevent damage to PSI reaction centers and reduce the production of harmful ROS in the thylakoid membrane [47]. CEF around PSI contributes to the generation of ΔpH by facilitating electron transfer from PSI to the plastoquinone pool and back to PSI through the Cyt *b*_*6*_*f* complex. This process is important for maintaining the balance between ATP and NADPH production by increasing pH levels to enhance ATP synthesis. Furthermore, the generation of ΔpH triggers qE to dissipate excess absorbed light energy [46]. In our study, we observed an upregulation of the *PGRL1* gene in sorghum plants under drought stress. This could be attributed to the down-regulation of ATP synthase, as an increase in the expression of CEF-related genes may compensate for the decrease in ATP synthesis. This process is particularly important for C4 metabolism, as the C4 metabolic cycle requires more energy than C3 photosynthesis [48]. Additionally, NPQ induction and PSII protection are closely linked to the essential function of PGR5/PGRL1-dependent CEF. An increased CEF rate leads to the formation of a ΔpH, which in turn results in increased NPQ. This process helps reduce the damage caused by excess light energy to PSII and prevents the overaccumulation of electrons at PSI [47]. The findings of our study on wheat regarding the expression of genes involved in the CEF pathway indicated a decrease in the expression of these genes, which suggests the inhibition of CEF in wheat. This inhibition is believed to result in photoinhibition of PSII and PSI due to disruption of the ROS scavenging system. This may explain why genes related to the reaction centers of these two photosystems are down-regulated in wheat, unlike in sorghum.

### 4.2 Dark reaction

Most of the essential photosynthetic processes, including the C3 cycle, LHC, and electron transport components, are shared between C3 and C4 photosynthesis. Therefore, it is reasonable to anticipate that these two photosynthetic pathways would generally demonstrate comparable responses to changes in water availability. However, there are notable differences between the two photosynthetic types, which could result in varying reactions to water stress at multiple levels [49]. The results of our investigation clearly demonstrated that the expression levels of genes participating in the Calvin cycle decreased significantly in sorghum and wheat leaves during drought stress.

The Calvin cycle, known as a photosynthetic carbon fixation cycle, assumes the vital task of converting CO_2_ into carbohydrates. Moreover, it facilitates the assimilation, transport, and utilization of photo-assimilates, which serve as organic byproducts of photosynthesis [30]. *RBCS* is an important gene involved in carbon fixation, that helps in the conversion of CO_2_ into ribulose 1, 5-bisphosphate, which is crucial for CO_2_ assimilation. Down-regulation of *RBCS* genes leads to a decrease in CO_2_ assimilation, consequently causing a decrease in the rate of photosynthesis [4]. Drought stress in plants causes a reduction in Rubisco activity, which in turn decreases the amount of carbon fixation during the initial phase of the Calvin cycle. Consequently, this reduction in carbon fixation subsequently lowers the demand for enzymes involved in the subsequent reactions within the Calvin cycle [50]. In this study, in addition to affecting *RBCS*, drought stress affected the expression of other genes encoding important Calvin cycle enzymes such as FBA, fructose-1,6- bisphosphatase (FBP), and triphosphate isomerase (TPI). The regeneration of ribulose-1,5-bisphosphate (RuBP) plays a vital role in determining the rate of photosynthesis and, consequently, the efficiency of photosynthetic carbon fixation. FBA primarily contributes to the regeneration of RuBP, thereby improving the overall efficiency of photosynthesis [51,52]. The expression of the *Rubisco*, *FBP*, and *TPI* significantly decreased during drought stress. This indicates that drought stress disrupts metabolic processes by inhibiting the carboxylation reaction of CO_2_ and preventing the regeneration of RuBP [52]. A coordinated decrease in the expression of Calvin cycle genes is expected, serving as a means of reducing energy wastage for unnecessary biomolecule synthesis [50].

C4 photosynthesis is a complex biological trait in plants that allows for increased biomass accumulation and adaptation to adverse environments through the addition of a CO_2_ concentration mechanism, resulting in improved efficiency in carbon assimilation, as well as higher water and nitrogen use efficiencies [53]. The key enzymes involved in C4 photosynthesis, namely, NADP–malic enzyme (NADP–ME), phosphoenolpyruvate carboxylase (PEPC), pyruvate, PPDK, and NADPmalic dehydrogenase (NADP–MDH), have evolved to concentrate CO_2_ for the Calvin cycle, particularly in dry and hot regions [54,55]. The orthologs of genes encoding classic C4 enzymes preexisted in their C3 ancestors. However, these genes are typically expressed at low levels in C3 plants. In contrast, in C4 plants, these genes are highly expressed and coregulated by various stimuli [56]. C4 photosynthesis-related genes have been detected in various C3 plants, such as wheat, rice, and soybean. In C3 plants, these genes are involved in nonphotosynthetic processes and contribute to drought tolerance [54]. PEPC has crucial functions in maintaining pH levels and replenishing tricarboxylic acid (TCA) cycle intermediates. NADPH, which is produced through reactions catalyzed by NADP-ME, plays a vital role in cell growth, proliferation, and detoxification [57]. During stress, plants need more NADPH to handle the increased production of reactive oxygen and nitrogen species, which can be harmful to the plant. The ascorbate-glutathione cycle, an antioxidative system, relies on NADPH to protect plants from oxidative stress. Additionally, NADPH production in the cytoplasm is crucial for plants grown under limited water supply for biosynthetic reactions like proline and mannitol production. Severe stress affecting water balance can lead to turgor loss and membrane damage, but NADP-ME plays a crucial role in repairing this damage by supplying pyruvate and NADPH for fatty acid biosynthesis. Additionally, NADP-ME has various other functions such as facilitating lignin biosynthesis, regulating cytosolic pH, controlling stomatal closure, and potentially contributing to drought avoidance and water conservation through manipulation of organic anion metabolism in guard cells [55,57,58].

MDH is crucial for the growth and development of plants, as well as their response to abiotic stress [59]. In our study, we observed upregulation of the *MDH* and *pMDH* genes in wheat, but no significant change was found in sorghum. plastidial NADP-dependent MDH (pdNADP-MDH) is an important enzyme in the malate valve, that releases extra reducing equivalents in chloroplasts as malate. On the other hand, pdNAD-MDH primarily functions in chloroplasts and nongreen plastids under darkness. PMDH, found in peroxisomes, plays a role in photorespiration and fatty acid β-oxidation [59]. The present study not only revealed changes in the expression of *NADP-ME* and *MDH* but also revealed a significant alteration in the expression of the *PPDK* gene in both wheat and sorghum plants. Notably, the expression pattern of this gene differed between the two plants, with an upregulation observed in wheat and a down-regulation in sorghum. PPDK has a pivotal function in the transport of amino acids, and its increased activity can significantly accelerate the mobilization of nitrogen and increase protein content [54]. During drought stress, the activity of PPDK increases, leading to the metabolism of pyruvate into PEP, which serves as the substrate for PEPC and the acceptor of CO_2_ [55]. Previous studies have shown that the activity of C4 photosynthesis-related enzymes (PEPC, NADP-ME, NADP-MDH, and PPDK) significantly increases in wheat under drought stress [54,60].

Our findings demonstrate that upregulation of C4 photosynthesis-related enzymes in wheat as a C3 plant may contribute to plant protection against drought stress. The expression of these enzymes may not only boost CO_2_ availability for the Calvin cycle, but more importantly, it may lead to increased NADPH production for the antioxidant system and the production of osmoprotectants such as proline, which also play a role in antioxidant defense. Therefore, wheat utilizes osmotic adjustment and antioxidative defense systems to adapt to stress. This adjustment may be related to the greater production of ROS during stress, which is more pronounced in C3 plants than in C4 plants due to their elevated photorespiration rate and decreased stress tolerance [12,54].

### 4.3 Porphyrin and chlorophyll metabolism

Tetrapyrroles are crucial components in a range of biological functions, such as photosynthesis and respiration. Four types of tetrapyrroles are present in higher plants: Chl, heme, siroheme, and phytochromobilin. Among these, Chls stands out as a tetrapyrrole compound containing Mg. They play a critical role in capturing light and facilitating energy conversion, directly influencing the process of photosynthesis in plants [28,61].

Chl metabolism is an intricate pathway that can be categorized into four separate pathways: the common pathway, the Mg and heme branches, the Chl cycle, and the Chl degradation pathway [62]. Chl biosynthesis is influenced by both external factors, such as light and temperature, and the activity of genes encoding enzymes involved in this process. In fact, any reduction in the activity of these enzymes can lead to the inhibition of Chl biosynthesis [29,61]. A significant number of genes encoding key enzymes involved in Chl biosynthesis were significantly affected at the transcriptional level under drought stress in both wheat and sorghum in our study (Fig. 5).

The synthesis of tetrapyrrole begins with glutamate, and the pathway to uroporphyrinogen III (Uro III) is shared by all classes of tetrapyrroles [63]. In this study, the genes responsible for the enzymes involved in this shared pathway were down-regulated in both wheat and sorghum. However, it is worth noting that the gene encoding sirohydrochlorin ferrochelatase (*SirB*; *At1g50170*, acc. no. in Arabidopsis), which plays a crucial role in the third step of siroheme biosynthesis from Uro III [64], was upregulated specifically in wheat (Fig. 5). This finding highlights the significance of this gene in ability of wheat to efficiently produce siroheme under stress. Siroheme plays a crucial role in regulating glutathione biosynthesis, which helps plants maintain their cellular redox state during environmental stress. Glutathione acts as an antioxidant, protecting plants from oxidative damage caused by various stresses, and serves as a substrate for metallothioneins, which help detoxify heavy metals in the cell cell [65]. Disruption of the biosynthesis pathway of siroheme results in the accumulation of light-sensitive intermediates derived from the Chl pathway. This, in turn, leads to the synthesis of ROS [66].

Both heme and Chl rely on the same synthesizers during the conversion of Uro III to protoporphyrin IX (Proto IX) [66]. The expression of these synthesizers decreased under stress in our study. PPO plays a crucial role in the biosynthesis of Chl and heme by catalyzing the conversion of protoporphyrinogen IX to Proto IX. This enzymatic reaction serves as the final shared step in the production of both heme and Chl. Proto IX, the product of this reaction, is a fundamental substrate for two enzymes, ferrochelatase (FC) and Mg-chelatase, at the tetrapyrrole biosynthesis branching point [67,68]. We observed upregulation of *FC*, a relatively simple and single subunit enzyme [69], in wheat under drought stress (Fig. 5). Studies have shown that salt stress can lead to an increase in heme levels in both rice and cucumber plants [66,70]. Increased heme levels and the up-regulation of *FC2* and *heme oxygenase* (*HO*) expression play significant roles in mitigating salt stress by providing the necessary cofactors for heme proteins. Moreover, in rice plants exposed to NaCl stress, an enzymatic ROS-scavenging system is activated. This activity, which may be influenced by elevated heme levels, aids in the detoxification of H_2_O_2_ during salt stress [70]. Heme accumulation negatively regulates Chl biosynthesis through feedback control of 5-aminolevulinic acid (ALA) synthesis, by inhibiting the activity of GluTR [65,71].

The initial committed stage in Chl biosynthesis is catalyzed by Mg-chelatase, a large enzyme consisting of four components. The components of Mg-chelatase include the H subunit (CHLH/ABAR), I subunit (CHLI), D subunit (CHLD), and GENOMES UNCOUPLED 4 (GUN4) proteins, which work together to regulate its activity in Chl biosynthesis [40,69]. The white leaf phenotype observed in many plants is a direct consequence of reduced Mg-chelatase activity, emphasizing the critical role these enzymes play in the regulation of Chl biosynthesis [72]. In our study, the expression levels of the *CHLH* and *CHLD* subunits decreased in wheat under drought conditions. However, in the case of sorghum, only *CHLD* decreased, and there were no significant changes in the other components of Mg-chelatase. Xie et al. [73] suggested a potential link between the down-regulation of *CHLH* and *CHLD* and the reduction in Chl *a* and *b* content in nanmu leaves under drought stress. CHLD and CHLI play a key role in regulating the transcription of photosynthesis-associated nuclear genes. Therefore, silencing *CHLD* and *CHLI* leads to a decrease in photosynthetic proteins. Additionally, Mg-chelatase not only is involved in tetrapyrrole biosynthesis but also influences ROS homeostasis, interorganellar metabolism, and retrograde signaling in plant cells [74]. The virus-induced gene silencing of *MeCHLD* in cassava leads to a decrease in Chl synthesis and photosynthesis by down-regulating genes involved in these processes, such as *RBCS*, *PEPC*, *CHLM*, and *CHLI*. Additionally, it affects starch accumulation in cassava storage roots, indicating its role in source-to-sink processes [75]. Zhao et al. [76] reported that drought stress has a negative impact on the synthesis of Chl in peony trees. The authors observed that key enzymes involved in porphyrin and Chl metabolism, such as CHLD, CHLI, CHLH, CRD, and HEMD, were inhibited under drought conditions. Our study on wheat revealed a down-regulation of genes encoding several enzymes involved in Chl biosynthesis, including CHLM, CRD, PPO, DVR, and protochlorophyllide oxidoreductase (POR). In the process of Chl biosynthesis, POR serves as a key reductase and is dependent on light in higher plants. Under dark conditions, inhibiting POR leads to the rapid accumulation of protochlorophyllide (Pchlide) and subsequently down-regulates ALA synthesis. This is due to a feedback regulatory mechanism between ALA and Pchlide synthesis synthesis [66].

During the final step of Chl synthesis, Chl *a* and Chl *b* undergo conversion through the Chl cycle [77]. Chl *a* is found in various protein complexes, such as PSI, PSII, and the cyt *b*_*6*_*f* complex. On the other hand, Chl *b* is exclusively present in the light-harvesting Chl *a/b*–protein complex [78]. Chl *b* is synthesized from Chl *a* through the enzymatic conversion of 7-hydroxymethyl Chl *a* by CAO [79]. The activity of the CAO is regulated through negative feedback mediated by Chl *b* [77]. Furthermore, the conversion of Chl *b* to Chl *a* is catalyzed in a two-step reaction by Chl *b* reductase (CBR) and hydroxymethyl chlorophyll *a* reductase (HCAR) [80]. In plants, two different isoforms of CBR have been identified. One isoform is encoded by the *NYC1* gene, while the other isoform is encoded by the *NOL* gene [79]. There are two functions of converting Chl *b* to Chl *a*: one is to regulate the ratio of Chl *a* to Chl *b*, and the other is to break down Chl *b*. This conversion is necessary because the enzyme PAO cannot catalyze the opening of the ring structure in pheophorbide *b* [77]. The overexpression of *CBR* accelerates leaf senescence and the degradation of Chl *b* [81]. Defects in *NYC1* or *NOL* result in the retention of Chl *b* and a stay-green phenotype [80]. Stay-green is an important drought-adaptation mechanism in cereals, especially sorghum. This trait allows sorghum to maintain grain filling even under drought stress, resulting in improved crop yield [82,83]. Knockdown of *LpNOL* has shown that NOL plays a crucial role in inducing the functional stay-green phenotype in perennial grass species by modulating various pathways related to Chl catabolism, photosynthesis, and other physiological processes [81]. On the other hand, it is well known that Chl *b* is the major pigment in LHCII that is involved in NPQ. A decrease in the amount of Chl *b* leads to lower levels of NPQ [84]. In our study, upregulation of *NYC1* and *NOL* was detected in wheat, which may be a sign of Chl degradation and subsequent LHCII degradation. However, in sorghum, the expression of the *NYC1*, *NOL*, and LHCII genes did not changed, which could indicate greater NPQ and a greater ability of this plant to cope with photoinhibition than wheat.

The breakdown of Chl is a necessary process in plants to protect cells from the harmful effects of phototoxic pigments and to recycle nitrogen from Chl-binding proteins during leaf senescence [30]. Several enzymes, including chlorophyllase (CLH), PPH, PAO, and red chlorophyll catabolite reductase (RCCR), are responsible for the degradation of Chl. CLH and PPH play a role in breaking down Chl molecules during the early stage of degradation, which can be induced by stress conditions to initiate leaf senescence [85]. Additionally, the protein SGR is required for the initiation of Chl breakdown and interacts with both Chl catabolic enzymes and LHCII [86]. It is believed that SGR destabilizes pigment-protein complexes to allow enzymes in the Chl breakdown pathway to access their substrate during leaf senescence [30]. The SGR family of higher plants has been classified into two groups: the SGR subfamily and the SGRL subfamily [86]. There are three *SGR* genes in *A. thaliana*, namely, *AtSGR1*, *AtSGR2*, and *AtSGRL* [87]. *SGR1* and *SGR2* highly expressed during senescence, whereas *SGRL* is primarily expressed in green tissues, and its expression is significantly decreased in senescent leaves. SGRL is involved in a reaction that converts chl *a* and Chlide *a* to pheophytin *a* and pheophorbide *a*, while SGR is responsible for converting chl *a* to pheophytin *a*. Neither subfamily uses chl *b* as a substrate, as Chl *b* tightly holds Mg, making it inaccessible for extraction by SGR [88]. Mutations in *SGR*, as well as mutations in *NYC1*, *NOL*, *HCAR*, *PPH*, and *PAO*, result in a stay-green phenotype in Arabidopsis. SGR-like plays a role in increasing the Chl content in carnation petals [72]. The activation of the Chl degradation pathway under drought stress was observed in both wheat and sorghum. However, the number of genes affected by stress was lower in sorghum. Additionally, the expression of the *NYC1* and *NOL* genes in sorghum did not significantly change in sorghum. Therefore, it appears that the activation of the Chl degradation pathway in sorghum is more focused on removing harmful intermediate molecules of the tetrapyrrole biosynthetic pathways.

Our results suggest that porphyrin and chlorophyll metabolism was affected by drought stress, possibly to prevent the accumulation of harmful singlet oxygen generating tetrapyrroles. In sorghum and wheat, this prevention was mediated by the feedback control of ALA synthesis by Pchlide. In wheat, in addition to Pchlide, heme also negatively regulated ALA synthesis and subsequently Chl metabolism. This may be the reason why more genes in the Chl metabolism pathway were differentially expressed under stress in wheat than in sorghum. In addition, the induction of heme and siroheme biosynthesis in wheat may have activated the antioxidant defense system as one of the plant adaptation mechanisms to stress, and thus protecting plants from oxidative damage caused by drought stress.

## 5 Limitation

In this study, the number of species studied was limited due to the orthology definition. Consequently, this limitation affects the generalizability of our findings, so we chose two species, wheat and sorghum, which are important cereal crops with high economic and agricultural value. Sorghum is also known as a drought-tolerant C4 plant, which makes it an attractive candidate for studying the impact of drought Therefore, understanding the response of sorghum to drought could be useful for future research to develop effective strategies to increase crop yield in C3 and C4 plants under challenging environmental conditions. Furthermore, our investigation was limited by its focus on the transcriptome level, and further research would be useful to fully understand the mechanisms and specific consequences of changes in the expression of the identified genes related to stress tolerance and plant physiology.

## 6 Conclusion

Drought stress has a significant impact on the process of photosynthesis in plants, resulting in a reduced photosynthesis rate and ultimately affecting plant growth and yield. It is crucial to understand the effects of drought stress on photosynthetic pathways, particularly the differences between C3 and C4 plants, to maximize agricultural productivity and maintain food security. By conducting a meta-analysis, we have clarified the intricate relationship between drought stress and the expression of photosynthesis-related genes in C3 and C4 plants. Our findings revealed the specific genes and components of the photosynthesis pathway that are affected by stress in wheat and sorghum plants. In sorghum, genes related to CEF respond to stress conditions by preventing photoinhibition and damage to photosystems, particularly PSI. In contrast, in wheat, increasing the expression of homologous genes of C4 enzymes likely helps the plant cope with stress by providing energy for the defense system. This suggests the important role of these enzymes in response to drought stress. This study provides insights into the function of photosynthesis-related genes under stress conditions and can be useful for future research to develop drought-tolerant varieties by improving C3 and C4 photosynthetic pathways and engineering C4 photosynthesis in crops, which will ultimately increase agricultural productivity and food security.

## CRediT authorship contribution statement

**Shima Karami:** Writing – original draft, Validation, Investigation, Formal analysis, Data curation. **Behrouz Shiran:** Writing – review & editing, Supervision, Project administration, Conceptualization. **Rudabeh Ravash:** Writing – review & editing, Supervision, Project administration, Methodology, Conceptualization.

## Data availability statement

Data included in the article/supplementary material is referenced in the article.

## Declaration of competing interest

The authors declare that they have no known competing financial interests or personal relationships that could have appeared to influence the work reported in this paper.

## References

[1] Januškaitienė I., Dikšaitytė A., Kunigiškytė J. (2022). Organic fertilizers reduce negative effect of drought in barely (C3) and millet (C4) under warmed climate conditions. Arch. Agron Soil Sci..

[2] Song Z., Wang L., Lee M., Yue G.H. (2023). The evolution and expression of stomatal regulators in C3 and C4 crops, implications on the divergent drought tolerance. Front. Plant Sci..

[3] Yang X., Lu M., Wang Y., Wang Y., Liu Z., Chen S. (2021). Response mechanism of plants to drought stress. Horticulturae.

[4] Waititu J.K., Zhang X., Chen T., Zhang C., Zhao Y., Wang H. (2021). Transcriptome analysis of tolerant and susceptible maize genotypes reveals novel insights about the molecular mechanisms underlying drought responses in leaves. Int. J. Mol. Sci..

[5] Fang Y., Xiong L. (2015). General mechanisms of drought response and their application in drought resistance improvement in plants. Cell. Mol. Life Sci..

[6] Uzilday B., Turkan I., Sekmen A.H., Ozgur R.E.N.G., Karakaya H.C. (2012). Comparison of ROS formation and antioxidant enzymes in *Cleome gynandra* (C4) and *Cleome spinosa* (C3) under drought stress. Plant Sci..

[7] Cohen I., Zandalinas S.I., Huck C., Fritschi F.B., Mittler R. (2021). Meta‐analysis of drought and heat stress combination impact on crop yield and yield components. Physiol. Plant..

[8] Stepien P., Klobus G. (2005). Antioxidant defense in the leaves of C3 and C4 plants under salinity stress. Physiol. Plant..

[9] Kulya J., Lontom W., Bunnag S., Theerakulpisut P. (2011). *Cleome gynandra* L. (C4 plant) shows higher tolerance of salt stress than its C3 close relative, *C. viscosa* L. Adv. Agric. Bot..

[10] Karami S., Shiran B., Ravash R., Fallahi H. (2023). A comprehensive analysis of transcriptomic data for comparison of plants with different photosynthetic pathways in response to drought stress. PLoS One.

[11] Zhang Q., Qi X., Xu W., Li Y., Zhang Y., Peng C., Fang Y. (2021). Response of transgenic Arabidopsis expressing maize C4 photosynthetic enzyme genes to high light. Plant Signal. Behav..

[12] Sonmez M.C., Ozgur R., Uzilday B., Turkan I., Ganie S.A. (2023). Redox regulation in C3 and C4 plants during climate change and its implications on food security. Food Energy Secur..

[13] Keel B.N., Zarek C.M., Keele J.W., Kuehn L.A., Snelling W.M., Oliver W.T., Freetly H.C., Lindholm-Perry A.K. (2018). RNA-Seq Meta-analysis identifies genes in skeletal muscle associated with gain and intake across a multi-season study of crossbred beef steers. BMC Genom..

[14] Corchete L.A., Rojas E.A., Alonso-López D., De Las Rivas J., Gutiérrez N.C., Burguillo F.J. (2020). Systematic comparison and assessment of RNA-seq procedures for gene expression quantitative analysis. Sci. Rep..

[15] Ranjbar R., Behzadi P., Mammina C. (2016). Respiratory tularemia: francisella tularensis and microarray probe designing. Open Microbiol. J..

[16] Behzadi P., Sameer A.S., Nissar S., Banday M.Z., Gajdács M., García-Perdomo H.A., Akhtar K., Pinheiro M., Magnusson P., Sarshar M., Ambrosi C. (2022). The Interleukin-1 (IL-1) superfamily cytokines and their single nucleotide polymorphisms (SNPs). J. Immunol. Res..

[17] Andrews S. (2010). http://www.bioinformatics.babraham.ac.uk/projects/fastqc/.

[18] Bolger A.M., Lohse M., Usadel B. (2014). Trimmomatic: a flexible trimmer for Illumina sequence data. Bioinformatics.

[19] Dobin A., Davis C.A., Schlesinger F., Drenkow J., Zaleski C., Jha S., Batut P., Chaisson M., Gingeras T.R. (2013). STAR: ultrafast universal RNA-seq aligner. Bioinformatics.

[20] Robinson M., McCarthy D. (2010). edgeR: differential expression analysis of digital gene expression data. Bioconductor.Fhcrc.Org.

[21] Rau A., Marot G., Jaffrézic F. (2014). Differential meta-analysis of RNA-seq data from multiple studies. BMC Bioinf..

[22] Ge S.X., Jung D., Yao R. (2020). ShinyGO: a graphical gene-set enrichment tool for animals and plants. Bioinformatics.

[23] Shannon P., Markiel A., Ozier O., Baliga N.S., Wang J.T., Ramage D., Amin N., Schwikowski B., Ideker T. (2003). Cytoscape: a software Environment for integrated models of biomolecular interaction networks. Genome Res..

[24] Zhao S., Zhang B., Zhang Y., Gordon W., Du S., Paradis T., Vincent M., von Schack D., Abdurakhmonov I.Y. (2016). Bioinformatics—updated Features and Applications.

[25] Tomczak A., Mortensen J.M., Winnenburg R., Liu C., Alessi D.T., Swamy V., Vallania F., Lofgren S., Haynes W., Shah N.H., Musen M.A., Khatri P. (2018). Interpretation of biological experiments changes with evolution of the Gene Ontology and its annotations. Sci. Rep..

[26] Liu C., Duan N., Chen X., Li H., Zhao X., Duo P., Wang J., Li Q. (2022). Metabolic pathways involved in the drought stress response of *Nitraria tangutorum* as revealed by transcriptome analysis. Forests.

[27] Wasilewska-Dębowska W., Zienkiewicz M., Drozak A. (2022). How light reactions of photosynthesis in C4 plants are optimized and protected under high light conditions. Int. J. Mol. Sci..

[28] Tanaka R., Tanaka A. (2007). Tetrapyrrole biosynthesis in higher plants. Annu. Rev. Plant Biol..

[29] Niu K., Ma H. (2018). The positive effects of exogenous 5-aminolevulinic acid on the chlorophyll biosynthesis, photosystem and calvin cycle of Kentucky bluegrass seedlings in response to osmotic stress. Environ. Exp. Bot..

[30] Wang Q.L., Chen J.H., He N.Y., Guo F.Q. (2018). Metabolic reprogramming in chloroplasts under heat stress in plants. Int. J. Mol. Sci..

[31] Szklarczyk D., Gable A.L., Lyon D., Junge A., Wyder S., Huerta-Cepas J., Simonovic M., Doncheva N.T., Morris J.H., Bork P., Jensen L.J., Mering C.V. (2019). STRING v11: protein-protein association networks with increased coverage, supporting functional discovery in genome-wide experimental datasets. Nucleic Acids Res..

[32] Cardona T., Shao S., Nixon P.J. (2018). Enhancing photosynthesis in plants, the light reactions. Essays Biochem..

[33] González M.C., Cejudo F.J., Sahrawy M., Serrato A.J. (2021). Current knowledge on mechanisms preventing photosynthesis redox imbalance in plants. Antioxidants.

[34] Alameldin H.F., Montgomery B.L. (2023). Plasticity of Arabidopsis rosette transcriptomes and photosynthetic responses in dynamic light conditions. Plant Direct.

[35] Zhao S., Gao H., Luo J., Wang H., Dong Q., Wang Y., Tao J. (2020). Genome-wide analysis of the light-harvesting chlorophyll *a/b*-binding gene family in apple (*Malus domestica*) and functional characterization of MdLhcb4. 3, which confers tolerance to drought and osmotic stress. Plant Physiol. Biochem..

[36] Daszkowska-Golec A., Collin A., Sitko K., Janiak A., Kalaji H.M., Szarejko I. (2019). Genetic and physiological dissection of photosynthesis in barley exposed to drought stress. Int. J. Mol. Sci..

[37] Yang M., Geng M., Shen P., Chen X., Li Y., Wen X. (2019). Effect of post-silking drought stress on the expression profiles of genes involved in carbon and nitrogen metabolism during leaf senescence in maize (*Zea mays* L.). Plant Physiol. Biochem..

[38] Cruz J.A., Avenson T.J., Kanazawa A., Takizawa K., Edwards G.E., Kramer D.M. (2005). Plasticity in light reactions of photosynthesis for energy production and photoprotection. J. Exp. Bot..

[39] Hashida S.N., Kawai-Yamada M. (2019). Inter-organelle NAD metabolism underpinning light responsive NADP dynamics in plants. Front. Plant Sci..

[40] Yuan L., Zhang L., Wu Y., Zheng Y., Nie L., Zhang S., Lan T., Zhao Y., Zhu S., Hou J., Chen G., Tang X., Wang C. (2021). Comparative transcriptome analysis reveals that chlorophyll metabolism contributes to leaf color changes in wucai (*Brassica campestris* L.) in response to cold. BMC Plant Biol..

[41] Sun Q., Wang T., Huang J., Gu X., Dong Y., Yang Y., Da X., Mo X., Xie X., Jiang H., Yan D., Zheng B., He Y. (2023). Transcriptome analysis reveals the response mechanism of *Digitaria sanguinalis, Arabidopsis thaliana* and *Poa annua* under 4, 8-Dihydroxy-1-tetralone treatment. Plants.

[42] Parrine D., Greco T.M., Muhammad B., Wu B.S., Zhao X., Lefsrud M. (2021). Color-specific recovery to extreme high-light stress in plants. Life.

[43] Wang C., Yamamoto H., Shikanai T. (2015). Role of cyclic electron transport around photosystem I in regulating proton motive force. Biochim. Biophys. Acta, Bioenerg..

[44] Zhang H., Xu Z., Guo K., Huo Y., He G., Sun H., Guan Y., Xu N., Yang W., Sun G. (2020). Toxic effects of heavy metal Cd and Zn on chlorophyll, carotenoid metabolism and photosynthetic function in tobacco leaves revealed by physiological and proteomics analysis. Ecotoxicol. Environ. Saf..

[45] Galmés J., Medrano H., Flexas J. (2007). Photosynthesis and photoinhibition in response to drought in a pubescent (var. minor) and a glabrous (var. palaui) variety of Digitalis minor. Environ. Exp. Bot..

[46] Yamori W. (2016). Photosynthetic response to fluctuating environments and photoprotective strategies under abiotic stress. J. Plant Res..

[47] Lu J., Yin Z., Lu T., Yang X., Wang F., Qi M., Li T., Liu Y. (2020). Cyclic electron flow modulate the linear electron flow and reactive oxygen species in tomato leaves under high temperature. Plant Sci..

[48] Chang L., Wang L., Peng C., Tong Z., Wang D., Ding G., Xiao J., Guo A., Wang X. (2019). The chloroplast proteome response to drought stress in cassava leaves. Plant Physiol. Biochem..

[49] Ghannoum O. (2009). C4 photosynthesis and water stress. Ann. Bot..

[50] Xue G.P., McIntyre C.L., Glassop D., Shorter R. (2008). Use of expression analysis to dissect alterations in carbohydrate metabolism in wheat leaves during drought stress. Plant Mol. Biol..

[51] Wang X., Liu H., Zhang D., Zou D., Wang J., Zheng H., Jia Y., Qu Z., Sun B., Zhao H. (2022). Photosynthetic carbon fixation and sucrose metabolism supplemented by weighted gene co-expression network analysis in response to water stress in rice with overlapping growth stages. Front. Plant Sci..

[52] Zhang A., Liu M., Gu W., Chen Z., Gu Y., Pei L., Tian R. (2021). Effect of drought on photosynthesis, total antioxidant capacity, bioactive component accumulation, and the transcriptome of *Atractylodes lancea*. BMC Plant Biol..

[53] Bräutigam A., Kajala K., Wullenweber J., Sommer M., Gagneul D., Weber K.L., Carr K.M., Gowik U., Mass J., Lercher M.J., Westhoff P., Hibberd J.M., Weber A.P. (2011). An mRNA blueprint for C4 photosynthesis derived from comparative transcriptomics of closely related C3 and C4 species. Plant Physiol..

[54] Zhang X., Pu P., Tang Y., Zhang L., Lv J. (2019). C4 photosynthetic enzymes play a key role in wheat spike bracts primary carbon metabolism response under water deficit. Plant Physiol. Biochem..

[55] Hýsková V.D., Miedzińska L., Dobrá J., Vankova R., Ryšlavá H. (2014). Phosphoenolpyruvate carboxylase, NADP-malic enzyme, and pyruvate, phosphate dikinase are involved in the acclimation of *Nicotiana tabacum* L. to drought stress. J. Plant Physiol..

[56] Ding Z., Weissmann S., Wang M., Du B., Huang L., Wang L., Tu X., Zhong S., Myers C., Brutnell T.P., Sun Q., Li P. (2015). Identification of photosynthesis-associated C4 candidate genes through comparative leaf gradient transcriptome in multiple lineages of C3 and C4 species. PLoS One.

[57] Doubnerová V., Ryšlavá H. (2011). What can enzymes of C4 photosynthesis do for C3 plants under stress?. Plant Sci..

[58] Guo P., Baum M., Grando S., Ceccarelli S., Bai G., Li R., von Korff M., Varshney R.K., Graner A., Valkoun J. (2009). Differentially expressed genes between drought-tolerant and drought-sensitive barley genotypes in response to drought stress during the reproductive stage. J. Exp. Bot..

[59] Li Z., Shi L., Lin X., Tang B., Xing M., Zhu H. (2023). Genome-wide identification and expression analysis of malate dehydrogenase gene family in sweet potato and its two diploid relatives. Int. J. Mol. Sci..

[60] Jia S., Lv J., Jiang S., Liang T., Liu C., Jing Z. (2015). Response of wheat ear photosynthesis and photosynthate carbon distribution to water deficit. Photosynthetica.

[61] Yu Q., Shen Y., Wang Q., Wang X., Fan L., Wang Y., Zhang S., Liu Z., Zhang M. (2019). Light deficiency and waterlogging affect chlorophyll metabolism and photosynthesis in *Magnolia sinostellata*. Trees (Berl.).

[62] Kong W., Yu X., Chen H., Liu L., Xiao Y., Wang Y., Wang C., Lin Y., Yu Y., Wang C., Jiang L., Zhai H., Zhao Z., Wan J. (2016). The catalytic subunit of magnesium-protoporphyrin IX monomethyl ester cyclase forms a chloroplast complex to regulate chlorophyll biosynthesis in rice. Plant Mol. Biol..

[63] Zhao Y., Xu W., Wang L., Han S., Zhang Y., Liu Q., Liu B., Zhao X. (2022). A maize necrotic leaf mutant caused by defect of coproporphyrinogen III oxidase in the porphyrin pathway. Genes.

[64] Tripathy B.C., Sherameti I., Oelmüller R. (2010). Siroheme: an essential component for life on earth. Plant Signal. Behav..

[65] Zhang Z.W., Zhang G.C., Zhu F., Zhang D.W., Yuan S. (2015). The roles of tetrapyrroles in plastid retrograde signaling and tolerance to environmental stresses. Planta.

[66] Wu Y., Liao W., Dawuda M.M., Hu L., Yu J. (2019). 5-Aminolevulinic acid (ALA) biosynthetic and metabolic pathways and its role in higher plants: a review. Plant Growth Regul..

[67] Li X., Volrath S.L., Nicholl D.B., Chilcott C.E., Johnson M.A., Ward E.R., Law M.D. (2003). Development of protoporphyrinogen oxidase as an efficient selection marker for Agrobacterium tumefaciens-mediated transformation of maize. Plant Physiol..

[68] Brzezowski P., Ksas B., Havaux M., Grimm B., Chazaux M., Peltier G., Johnson X., Alric J. (2019). The function of PROTOPORPHYRINOGEN IX OXIDASE in chlorophyll biosynthesis requires oxidised plastoquinone in Chlamydomonas reinhardtii. Commun. Biol..

[69] Farmer D.A., Brindley A.A., Hitchcock A., Jackson P.J., Johnson B., Dickman M.J., Hunter C.N., Rei J.D., Adams N.B. (2019). The ChlD subunit links the motor and porphyrin binding subunits of magnesium chelatase. Biochem. J..

[70] Nguyen A.T., Tran L.H., Jung S. (2023). Salt stress-induced modulation of porphyrin biosynthesis, photoprotection, and antioxidant properties in rice plants (*Oryza sativa*). Antioxidants.

[71] Nott A., Jung H.S., Koussevitzky S., Chory J. (2006). Plastid-to-nucleus retrograde signaling. Annu. Rev. Plant Biol..

[72] Ohmiya A., Hirashima M., Yagi M., Tanase K., Yamamizo C. (2014). Identification of genes associated with chlorophyll accumulation in flower petals. PLoS One.

[73] Xie N., Li B., Yu J., Shi R., Zeng Q., Jiang Y., Zhao D. (2022). Transcriptomic and proteomic analyses uncover the drought adaption landscape of *Phoebe zhennan*. BMC Plant Biol..

[74] Luo T., Luo S., Araújo W.L., Schlicke H., Rothbart M., Yu J., Fan T., Fernie A.R., Grimm B., Luo M. (2013). Virus-induced gene silencing of pea *CHLI* and *CHLD* affects tetrapyrrole biosynthesis, chloroplast development and the primary metabolic network. Plant Physiol. Biochem..

[75] Yang X., Cai J., Xue J., Luo X., Zhu W., Xiao X., Xue M., An F., Li K., Chen S. (2023). Magnesium chelatase subunit D is not only required for chlorophyll biosynthesis and photosynthesis, but also affecting starch accumulation in Manihot esculenta Crantz. BMC Plant Biol..

[76] Zhao D., Zhang X., Fang Z., Wu Y., Tao J. (2019). Physiological and transcriptomic analysis of tree peony (*Paeonia section* Moutan DC.) in response to drought stress. Forests.

[77] Jia T., Ito H., Hu X., Tanaka A. (2015). Accumulation of the NON‐YELLOW COLORING 1 protein of the chlorophyll cycle requires chlorophyll *b* in *Arabidopsis thaliana*. Plant J..

[78] Kusaba M., Ito H., Morita R., Iida S., Sato Y., Fujimoto M., Hirochika H., Nishimura M., Tanaka A. (2007). Rice NON-YELLOW COLORING1 is involved in light-harvesting complex II and grana degradation during leaf senescence. Plant Cell.

[79] Tanaka A., Tanaka R., Grimm B. (2019).

[80] Jibran R., Sullivan K.L., Crowhurst R., Erridge Z.A., Chagné D., McLachlan A.R., Brummell D.A., Dijkwel P.P., Hunter D.A. (2015). Staying green postharvest, how three mutations in the Arabidopsis chlorophyll *b* reductase gene NYC1 delay degreening by distinct mechanisms. J. Exp. Bot..

[81] Yu G., Xie Z., Zhang J., Lei S., Lin W., Xu B., Huang B. (2021). NOL‐mediated functional stay‐green traits in perennial ryegrass (*Lolium perenne* L.) involving multifaceted molecular factors and metabolic pathways regulating leaf senescence. Plant J..

[82] Borrell A.K., Mullet J.E., George-Jaeggli B., van Oosterom E.J., Hammer G.L., Klein P.E., Jordan D.R. (2014). Drought adaptation of stay-green sorghum is associated with canopy development, leaf anatomy, root growth, and water uptake. J. Exp. Bot..

[83] Velazco J.G., Jordan D.R., Mace E.S., Hunt C.H., Malosetti M., Van Eeuwijk F.A. (2019). Genomic prediction of grain yield and drought-adaptation capacity in sorghum is enhanced by multi-trait analysis. Front. Plant Sci..

[84] Turowski V.R., Aknin C., Maliandi M.V., Buchensky C., Leaden L., Peralta D.A., Busi, Araya A., Gomez-Casati D.F. (2015). Frataxin is localized to both the chloroplast and mitochondrion and is involved in chloroplast Fe-S protein function in Arabidopsis. PLoS One.

[85] Gan L., Han L., Yin S., Jiang Y. (2020). Chlorophyll metabolism and gene expression in response to submergence stress and subsequent recovery in perennial ryegrass accessions differing in growth habits. J. Plant Physiol..

[86] Sakuraba Y., Park S.Y., Paek N.C. (2015). The divergent roles of STAYGREEN (SGR) homologs in chlorophyll degradation. Mol. Cell..

[87] Shimoda Y., Ito H., Tanaka A. (2016). Arabidopsis STAY-GREEN, Mendel's green cotyledon gene, encodes magnesium-dechelatase. Plant Cell.

[88] Wang P., Grimm B. (2021). Connecting chlorophyll metabolism with accumulation of the photosynthetic apparatus. Trends Plant Sci..

