# Supplementary material for: Molecular investigation of how drought stress affects chlorophyll metabolism and photosynthesis in leaves of C3 and C4 plant species: A transcriptome meta-analysis
Source: Heliyon. 2025 Jan 29;11(3):e42368. doi: 10.1016/j.heliyon.2025.e42368 (PMC11840503; doi:10.1016/j.heliyon.2025.e42368)
Supplement: Multimedia component 1 [file mmc1.docx]

Supplementary Material

# Supplementary Tables

# Supplementary Table S1. Differentially expressed genes involved in photosynthetic processes in wheat under drought stress.

# Supplementary Table S2. Differentially expressed genes involved in photosynthetic processes in sorghum under drought stress.
